# Heat Shock Protein 70 Family in Response to Multiple Abiotic Stresses in the Silkworm

**DOI:** 10.3390/insects12100928

**Published:** 2021-10-12

**Authors:** Shou-Min Fang, Qian Zhang, Yu-Li Zhang, Gui-Zheng Zhang, Ze Zhang, Quan-You Yu

**Affiliations:** 1 School of Life Sciences, Chongqing University, Chongqing 400044, China; fangshoumin@126.com (S.-M.F.); zhangqiankobe24@163.com (Q.Z.); zezhang@cqu.edu.cn (Z.Z.); 2 College of Life Science, China West Normal University, Nanchong 637002, China; 3 Guangxi Academy of Sericultural Sciences, Nanning 530007, China; ZYL8324@126.com (Y.-L.Z.); zhangdoudou1999@163.com (G.-Z.Z.)

**Keywords:** heat shock protein 70, genome-wide, stress response, silkworm

## Abstract

**Simple Summary:**

Heat shock protein 70 family is widely distributed in all the organisms, which plays important roles in protein folding and preventing protein denaturation. Heat or cold stress response has been studied in some insects, but there is a lack of systematic investigation on the response of the same species to multiple stressors. Here, we performed genome-wide identification of heat shock protein 70 family in the silkworm, *Bombyx mori*. Using the silkworm as a model, the transcription profiles of all the genes against heat, cold, and pesticides were studied. Our findings would provide insights into the functional diversification of heat shock proteins 70 in insects.

**Abstract:**

The 70 kDa heat shock proteins play important roles in protecting organisms against environmental stresses, which are divided into stress-inducible forms (HSP70s) and heat shock cognates (HSC70s). In this study, heat shock protein 70 family was identified in the whole genome of the silkworm. Based on the known nomenclature and phylogenetic analysis, four HSP70s and five HSC70s were classified. Relatively, heat shock cognates were more conservative and were constitutively expressed in various tissues of the silkworm larvae. Under thermal (37 °C and 42 °C) and cold (2 °C) stresses, the expressions of *HSP70–1*, *HSP70–2*, and *HSP70–3* were up-regulated, and the highest induction reached 4147.3, 607.1, and 1987.3 times, respectively. Interestingly, *HSC70–1*, *HSC70–4*, and *HSC70–5* also showed slight induced expressions in the fat body and/or midgut under thermal stresses. In addition, the expression of *HSP70–1* was induced by dichlorvos and phoxim insecticides, while most HSC70 genes were inhibited. The results suggested that stress-inducible forms play more important roles in adaptation to various stresses than HSC70s.

## 1. Introduction

Heat shock proteins (HSPs) are originally identified as stress-responsive proteins, which are considered to play important roles in thermal/cold adaptation and other proteotoxic tolerances [1,2,3,4]. Heat shock proteins 70, approximately 70 kDa in molecular weight, are found in all organisms [5,6,7]. Heat shock protein 70 family includes stress-inducible genes (HSP70s) and constitutively expressed members or heat shock cognates (HSC70s). Inducible HSP70s help proteins obtain a natural conformation after partial denaturation during environmental stress, HSC70s participate in various processes in an unstressed cell, such as folding of proteins after translation or membrane translocation [1,8].

Stress-inducible HSP70s are universal tools of cellular protein folding and have long been considered as one of the key parts involved in heat stress adaptation and resistance [9,10]. Up-regulations of *DmHSP68*, *DmHSP70A*, and *DmHSP70Ba* were associated with an increase of thermal tolerance in *Drosophila melanogaster* after 36 °C heat shock for 1 h [11]. The heat shock tolerance of *Bactrocera dorsalis* was significantly enhanced by heat hardening at 35, 37, 39, and 41 °C, which was suggested that species with a wider distribution are more plastic than those with a narrower distribution [12]. In addition, up-regulation of HSP70 mRNA in response to high temperature was also observed in other organisms, such as *Bemisia tabaci* [13], *D. buzzatii* [14], *Anopheles gambiae* [15], *Chrysome aeneicollis* [16], *Nilaparvata lugens* [17]. Thus, up-regulation of HSP70 expression should be one of the adaptive mechanisms to thermal stress.

In insects, HSP70 proteins play an important role in coping with low-temperature stress [18,19,20]. The inducible response of HSP70 proteins was firstly found in *D. melanogaster* after a cold treatment [21]. *DmHSP68* and *DmHSP70Aa* were the most notable induced genes after cold (0 °C) treatment, and their expressions reached the peaks after 2 h of recovery at 25 °C [22]. In *Pyrrhocoris apterus*, one stress-inducible member (*PaHSP70*) and one cognate form (*PaHSC70*) were cloned [23]. *PaHSP70* was significantly up-regulated in response to low-temperature stimuli, while no up-regulation of *PaHSC70* was apparent after cold stress [23]. The direct evidence for a positive role of HSP70 in cold tolerance was verified in *Sarcophaga crassipalpis*, which was found that cold tolerance was decreased dramatically after injection of double-strand RNA of *ScHSP70* into the pre-diapause larvae [18].

HSP70s might be related to cellular protection against insecticide and other xenobiotic stresses [24,25]. The expression of HSP70 mRNA in the testes of *D. melanogaster* increased significantly when exposed to 0.15–1.5 ppb dichlorvos [24,26]. Significant up-regulation of an HSP70 gene was observed in *Cyprinus carpio* with chlorpyrifos and the binary mixture of atrazine/chlorpyrifos [27]. The up-regulation of HSP70s may be induced by oxidative stress caused by insecticide exposure [27,28], which plays an important role in promoting the correct folding of stress-accumulated misfolded proteins and preventing the formation of protein aggregates [29]. It was suggested that HSP70s are valuable biomarkers used to monitor the impact of insecticide and other environmental toxicants [25,30,31].

With the development of genome sequencing technology, large amounts of insect genomes have been released. In recent years, HSP70/HSC70 family has also been identified in the whole genomes of some species [13,32,33]. However, there is a lack of systematic investigation on response to multiple stressors in the same species. In this study, genome-wide identification of the HSP70/HSC70 family was conducted in *B. mori*. Phylogenetic relationship and sequence conservation were revealed. Especially, the stress responses of all the HSP70s/HSC70s were detected in the silkworm after exposure to thermal, cold, and three widely utilized organophosphorus insecticides (OPs). The results could provide valuable information for further functional characterization in insects. 2. Materials and Methods

## 2. Materials and Methods

### 2.1. Genome-Wide Identification of HSP70/HSC70 Genes in the Silkworm

The genome and annotation datasets of the silkworm were downloaded from RefSeq (Assembly accession: GCF_000151625.1) in NCBI (https://www.ncbi.nlm.nih.gov/ (accessed on 20 May 2020)). The known HSP70/HSC70 protein sequences (Table S1) of *D. melanogaster* [34] were used as queries to perform BLASTP search (*E*-value < 0.01) against the predicted protein database in the silkworm. Simultaneously, the Hidden Markov Model (PF00012) of heat shock proteins 70 was downloaded from the PFAM database (http://pfam.xfam.org/ (accessed on 1 May 2021)), which was used to screen the candidate HSP70s/HSC70s using Hmmsearch in HMMER 3.0 [35] with *E*-value < 0.01. Based on the BLASTP and Hmmsearch results, all the candidate HSP70s/HSC70s were checked by Hmmscan against PFAM (*E*-value < 1 × 10^−5^). In addition, all the checked HSP70s/HSC70s were used as queries to perform a TBLASTN (*E*-value < 0.01) search in the whole genome of the silkworms. The genomic intervals with its flanking region (1 kb or longer) of novel gene sites were extracted and predicted genes using Fgenesh+ (http://www.softberry.com/ (accessed on 10 June 2021)) [36]. The predicted genes were subsequently checked by Hmmscan against PFAM (*E*-value < 1 × 10^−5^). All the identified genes were used for further analysis.

### 2.2. Phylogenetic Analysis

The amino acid sequences of HSP70s/HSC70s were aligned using ClustalX v1.83 [37]. Positions that had a high percentage of gaps (>70%) were manually deleted. A phylogenetic tree was reconstructed with the maximum-likelihood method implemented in IQ-TREE v2.0.3 [38], using the “AUTO” amino acid substitution model and the ultrafast bootstrap (UFBoot) option with 1000 replicates. The best-fit model (LG+R4) was used for phylogenetic tree reconstruction. FigTree v1.4.4 (http://tree.bio.ed.ac.uk/software/figtree/ (accessed on 22 September 2021)) was used for tree visualization.

### 2.3. Silkworm Rearing and Stress Treatments

The silkworm, *Bombyx mori* L. (Lepidoptera: Bombycidae), was an important model organism of Lepidoptera [39], with strain *Dazao* as the best known at sericulture and genomics. This strain was reared at laboratory conditions, 25 ± 1 °C, 70% ± 2% relative humidity (RH), and photoperiod of 14 h light and 10 h dark. The rearing was maintained in mulberry leaves that were freshly collected from Plant Garden of Chongqing University. Leaves were washed with tap water before introducing them in the rearing cages.

Cold or thermal stress was performed by placing fifth instar 3-days old larvae at 2 °C, 37 °C, or 42 °C for 3 h, 6 h, and 9 h, allowing 2 h of recovery at 25 °C prior to dissection. Five individuals of each thermal and cold treatment were dissected, and their fat bodies, midguts or silk glands pooled per treatment to conform a sample. Each sample was stored at −80 °C after snap-frozen in liquid nitrogen till RNA extraction. Stress treatments were performed in triplicate.

### 2.4. Experimental Exposure to Insecticides

Technical-grade samples of chlorpyrifos, dichlorvos, and phoxim were purchased from Sigma-Aldrich (Shanghai) Trading Co, Ltd. (Shanghai, China). Stock solutions of chlorpyrifos, dichlorvos, and phoxim were prepared in reagent grade acetone at 50 mg/L, 2000 mg/L, and 20 mg/L, respectively. These stocks solutions were diluted in water to obtain the corresponding working solutions at 0.5 mg/L and 1.0 mg/L for chlorpyrifos, 40.0 and 80.0 mg/L for dichlorvos, and 0.375 and 0.75 mg/L for phoxim [40]. Fresh mulberry leaves were harvested from Plant Garden of Chongqing University, washed in tap water, air-dried for 60 min, and dipped into the corresponding insecticide working solution for 20 s. Treated leaves were allowed to dry at room temperature for 60 min, to allow solvent evaporation. The fifth instar 3-days old larvae were allowed to eat treated leaves for 24, 48, or 72 h, depending on the treatment. After exposure, 5 individuals of each treatment were dissected, isolated midguts, and fat bodies were stored separately at −80 °C after being snap-frozen in liquid nitrogen, till RNA extraction. Each treatment was performed in triplicate.

### 2.5. RNA Extraction and RT-PCR

Total RNA was extracted from each sample of midguts or fat bodies using TRIZOL reagent (Invitrogen, Carlsbad, CA, USA), following the manufacturer’s instructions. RNA concentration of each sample was determined by Nanovue spectrophotometry (GE Healthcare Life Sciences, Piscataway, NJ, USA), and all samples were subjected to RNase-free DNase I (Promega, Fitchburg, WI, USA) treatment to remove co-extracted DNA following instructions. For each sample, the first cDNA strand was obtained with M-MLV Reverse transcriptase (Promega, USA) using 1.5 µg of total RNA, following manufacturer’s instructions. RT-PCR primers were designed with Premier Primer 5.0 software (Table S2). The silkworm cytoplasmic *actin3* gene was used as an internal control. RT-PCR amplification was conducted in a final reaction volume of 25 µL containing 1 µL cDNA, 0.4 µM of each primer, 0.2 mM dNTPs, 1× EasyTaq buffer, and 2.5 units of EasyTaq DNA polymerase (Beijing TransGen Biotech, Beijing, China). PCRs were carried out with the following cycles: initial denaturation at 94 °C for 4 min; followed by 28 cycles of 30 s at 95 °C, 30 s annealing at a certain temperature (Table S2), 40 s extension (72 °C), and a final extension at 72 °C for 10 min. Amplification products were analyzed on 1.5% agarose gels.

### 2.6. Real-Time Quantitative PCR

Relative expression of HSP70/HSC70 genes was performed using CFX96™ Real-time PCR Detection System (Bio-Rad, Hercules, CA, USA). Each reaction was carried out in a total volume of 10 µL using 1 µL cDNA template, 5 µL SYBR^®^ *Premix Ex Taq*^TM^ II (TaKaRa, Osaka, Japan), and 0.4 µM of each gene-specific primer (Table S2). After 30 s at 95 °C, the cycling conditions were as follows: 39 cycles at 95 °C for 10 s, 55 °C for 30 s, and 95 °C for 10 s. The protocol ended with a melting program. Based on the comparative cycle threshold (Ct), relative expression was analyzed using the classic 2^−△△Ct^ method [41]. The ratio of target gene means expression in treated samples versus matched controls, which was normalized using the housekeeping reference gene (*actin3*). The mean relative expression was presented as Log_2_ ratio (i.e., Log_2_ mRNA abundance relative controls) [22].

### 2.7. Statistical Analysis

All analyses were performed using SPSS software, version 19.0 (SPSS, Chicago, IL, USA). The results were expressed as mean ± standard error of the mean (SEM). The difference in a mean between control and treatment was analyzed using one-way ANOVA. The *p*-value of < 0.05 was considered to demonstrate statistically significant differences.

## 3. Results

### 3.1. Identification of the HSP70/HSC70 Genes in the Silkworm Genome

**Figure 1:**
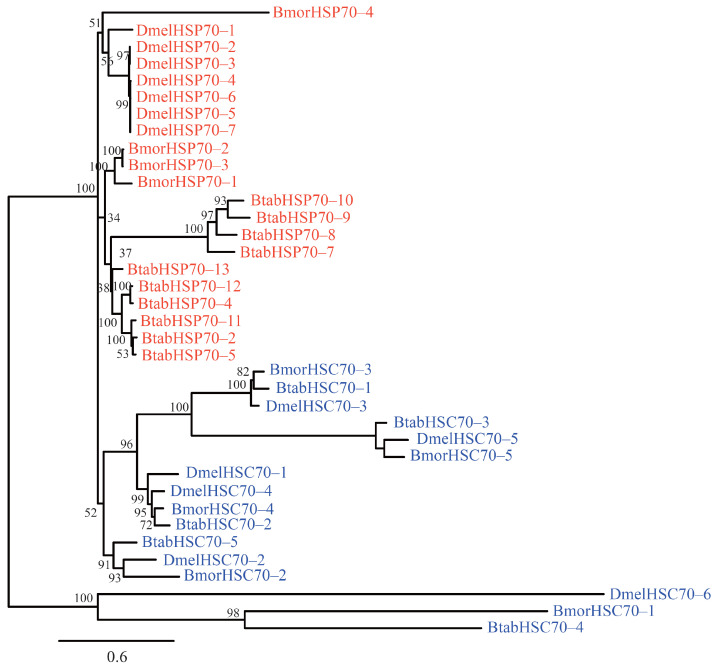
Maximum-likelihood phylogenetic tree of the HSP70/HSC70 proteins. The bootstrap values were displayed on the nodes. *Bmor*: *Bombyx mori*; *Dmel*: *Drosophila melanogaster*; *Btab*: *Bemisia tabaci*. Names of HSC70s were displayed in blue, all the HSP70s were displayed in red.

Based on BLASTP, Hmmsearch, and TBLASTN analyses, nine genes from the HSP70/HSC70 gene family were identified in the silkworm whole genome (Figure 1, Table S1). HSP70/HSC70 proteins have been well studies in *D. melanogaster* and *Bemisia tabaci* [13,34]. These two species were used for comparison with the silkworm. It should be noted that 17 putative genes were identified in *B. tabaci* [13]. However, the molecular weight of *BtabHsp70–1* was reached to 90.7 kDa, and *BtabHSP70–5* and *BtabHSP70–6* were isoforms of the heat shock protein 68-like gene (GeneID: 109039224). Thus, only 15 genes of *B. tabaci* were used in our analysis (Table S1). The maximum-likelihood phylogenetic tree was reconstructed for all the HSP70/HSC70 proteins of the silkworm and two model species (Figure 1). According to the nomenclature of *B. tabaci* and *D. melanogaster* [13,34], the name of each HSP70/HSC70 protein was assigned (Table S1). In this study, four stress-inducible forms (HSP70s) and five heat shock cognates (HSC70s) were identified in the silkworm. Interestingly, most HSC70s showed orthologous relationships in different species (Figure 1). For instance, *BmorHSC70–2*, *DmelHSC70–2*, and *BtabHSC70–5* were located in an independent phylogenetic clade, and the protein sequence identities were reached 72.61–80.10%. Thus, HSC70s were more conserved during evolution.

### 3.2. Conserved Signature Sequences and Typical Regions

**Figure 2:**
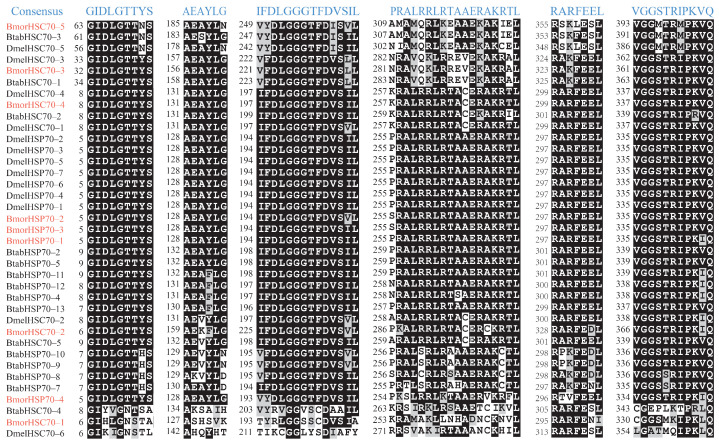
Signature sequences and typical regions of the heat shock proteins 70 family in *B. mori*, *D. melanogaster*, and *B. tabaci*. The consensus sequences were shown on the top of the alignments. The number before alignment represents the starting position in the corresponding sequence. The silkworm HSP70s/HSC70s were displayed in red. The similarity of the alignments was shaded with BOXSHADE v3.21 (https://embnet.vital-it.ch/software/BOX_form.html (accessed on 7 September 2021)).

Previous studies suggested that HSP70/HSC70 proteins have some characteristic sequences or motifs [42]. To understand the conservation of the characteristic sequences, all the HSP70/HSC70 proteins of *B. mori*, *B. tabaci*, and *D. melanogaster* were aligned by ClustalX v1.83 [37]. The consensus sequences [42] were used to locate the signatures and motifs, and the characteristic alignments were presented in Figure 2. Three signature sequences (‘GIDLGTTYS’, ‘IFDLGGGTFDVSIL,’ and ‘VGGSTRIPKVQ’) were found (Figure 2). Three other typical motifs were also identified, including an ATP-GTP binding site ‘AEAYLG’, a bipartite nuclear localization signal ‘PRALRRLRTAAERAKRTL’, and a non-organellar consensus motif ‘RARFEEL.’ These signatures and motifs were highly conserved in most of the silkworm HSP70/HSC70 proteins. The exception was that the three signature sequences, ATP-GTP binding site and bipartite nuclear localization signal presented diversification in *BmorHSC70–1*, *DmelHSC70–6*, and *BmorHSP70–4* (Figure 2). Whether the differentiation of these characteristic motifs affects their functions is worthy of further study.

### 3.3. Spatio-Temporal Expression Profiles of HSP70/HSC70 Genes in the Silkworm

**Figure 3:**
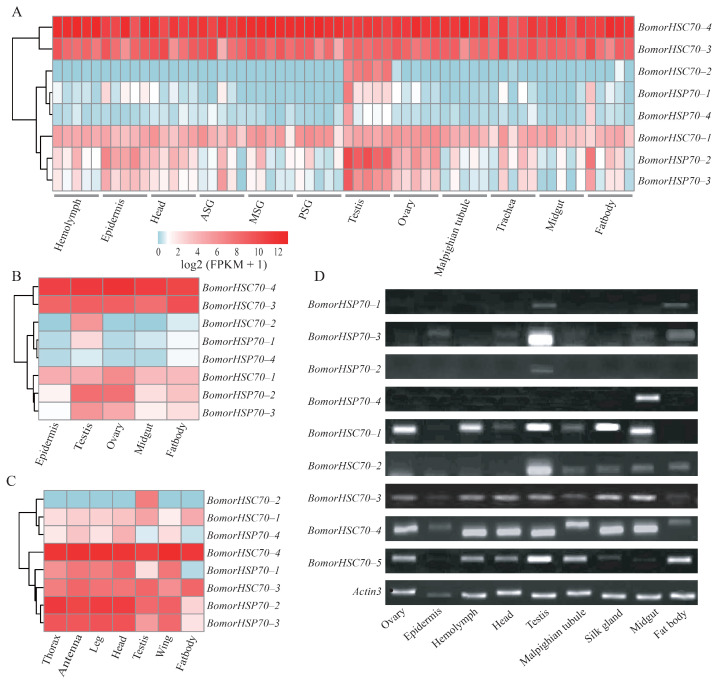
Spatio-temporal expression patterns of HSP70/HSC70 genes in the silkworm. (**A**) Expressions of HSP70/HSC70 genes in various tissues on day 3 of the fifth-instar larvae. (**B**) Transcriptional levels in various tissues of the pupae. (**C**) Tissue expression patterns in adults. Based on the transcriptome data, genome-wide analysis of developmental and tissue expression profiles was conducted in the silkworm and was deposited in SilkDB3.0 [43]. In this study, we downloaded the expression data (https://silkdb.bioinfotoolkits.net/base/download/ (accessed on 1 July 2021)) and extracted the corresponding expressions for the silkworm HSP70/HSC70 genes. Eight of them have expression data and were presented in (**A**–**C**). (**D**) Tissue expressions were detected by RT-PCR on day 3 of the fifth-instar larvae. The silkworm cytoplasmic actin *A3* gene was used as an internal control and abbreviated as *actin3*.

Expression profiles of HSP70/HSC70 genes were investigated in the various tissues and developmental stages of the silkworm (Figure 3). Based on the transcriptome analysis [43], 8 HSP70/HSC70 genes had corresponding transcriptional data (Figure 3A–C). It was indicated that *HSC70–1*, *HSC70–3*, and *HSC70–4* were highly expressed in almost all the tissues of larvae, pupae, and adults. *HSC70–2* showed specific expression in testis at all the developmental stages, implying that it may play an important role in spermatogenesis. For the stress-inducible forms, *HSP70–2* and *HSP70–3* showed constitutive expressions in most of the tissues of all three developmental stages. *HSP70–1* and *HSP70–4* were mainly expressed in adults, and *HSP70–1* also showed specific low expressions in the testis of the larvae and pupae. Expression profiles of all the 9 genes have been detected by RT-PCR in the various tissues on day 3 of the fifth-instar larvae (Figure 3D). RT-PCR results were similar to the transcriptome data (Figure 3A). In general, most of the heat shock cognates (HSC70s) presented constitutive expressions in all the tissues and developmental stages. Interestingly, some of the stress-inducible HSP70 genes were also constitutively expressed in various tissues of the pupae and adults (Figure 3B,C).

### 3.4. Expressions of HSP70s/HSC70s in Response to Thermal and Cold Stresses

**Figure 4:**
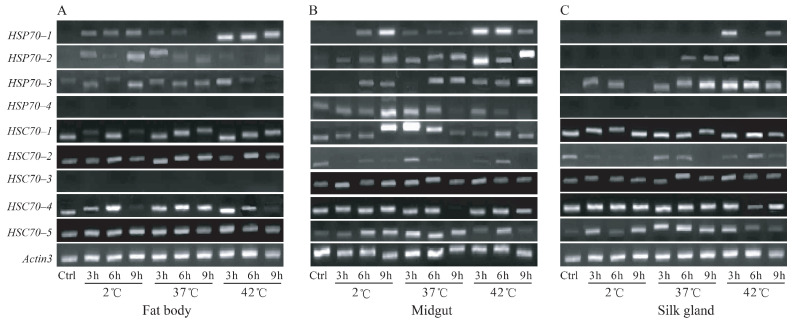
The transcriptional responses of *HSP70/HSC70* genes after cold and thermal exposures. (**A**–**C**) Gene responses in the fat body, midgut, and silk gland, respectively. The larvae on day 3 of the fifth instar were exposed to 2 °C for 3 h, 6 h, and 9 h, and heat shock at 37 °C and 42 °C for 3 h, 6 h, and 9 h, respectively. All the treatment followed by 2 h recovery at 25 °C. Ctrl: control.

**Figure 5:**
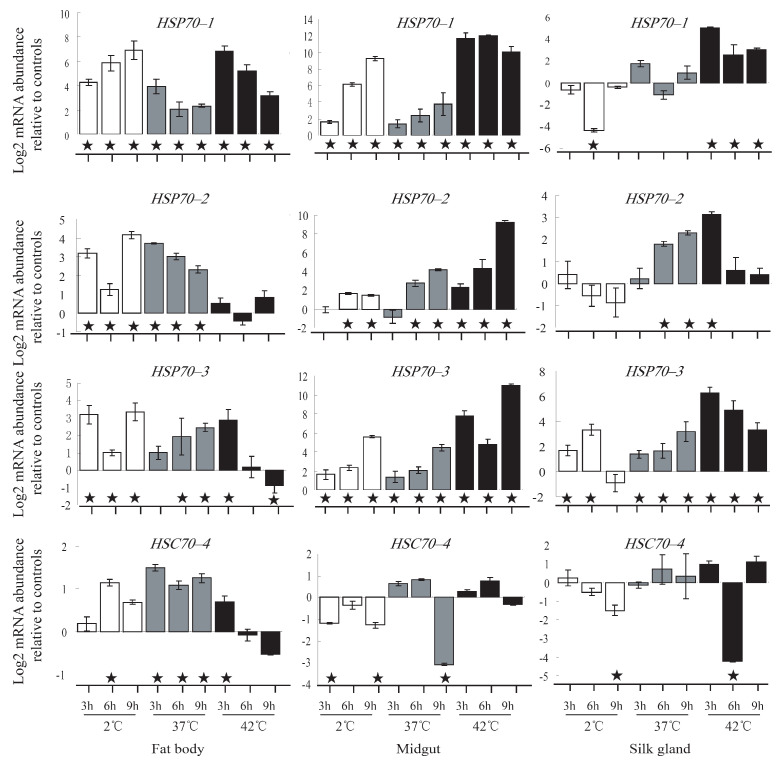
Relative expressions of *HSP70–1, HSP70–2*, *HSP70–3*, and *HSC70–4* in the silkworm after exposure to cold and thermal stresses. It was indicated by Log_2_ qPCR ratios (i.e., Log_2_ mRNA abundance relative controls). The symbol (★) indicates mean values that are significantly (*p* < 0.05) different between a treated sample and its corresponding control. The positive and negative values indicate up-regulation and down-regulation in the treatment, respectively.

After exposure to thermal stress, three representative tissues (fat body, midgut, and silk gland) were dissected in the silkworm. Transcriptional responses of the 9 *HSP70/HSC70* genes were investigated by RT-PCR and qPCR. The results indicated that *HSP70–1*, *HSP70–2*, and *HSP70–3* showed inducible responses to high-temperature stimulation in the fat body, midgut, and silk gland (Figure 4). The induced response was also found for the *HSC70–1* in the midgut, *HSC70–4* in the fat body, *HSC70–5* in the silk gland, respectively. The qPCR was conducted for some of the induced genes (Figure 5). The highest induction expressions of *HSP70–1*, *HSP70–2*, and *HSP70–3* were reached 4147.3, 607.1, and 1987.3 folds relative to the control, respectively (Figure 5). The induced response of *HSC70–4* was found only in the fat body, and its highest induction was only 7.5 times that of the control.

After exposure to cold stress, the expressions of *HSP70–1*, *HSP70–2*, and *HSP70–3* were also induced (Figure 4 and Figure 5). In the fat body and midgut, *HSP70–1* was the most cold-inducible gene, which the highest induction level was reached 621.5 times (Figure 5). Furthermore, the induction expression of *HSP70–1* was gradually improved with the increase of treatment time. The highest induction of *HSP70–2* and *HSP70–3* was only 48.8 and 17.6 folds, respectively (Figure 5). For the cognate forms (HSC70s), expression of *HSC70–2* was inhibited in the midgut and silk gland, while *HSC70–5* was induced in the two tissues (Figure 4B,C). Expression of *HSC70–4* was also detected by qPCR, which its induction was found at 6 h after treatment in the fat body (Figure 5). In general, whether it is cold or thermal stimulation, HSP70s showed higher inducible responses than HSC70s. These results suggested that the inducible forms (HSP70s) might play more important roles in thermal and cold stresses.

### 3.5. Stress Responses of HSP70s/HSC70s in the Silkworm under OPs Exposure

**Figure 6:**
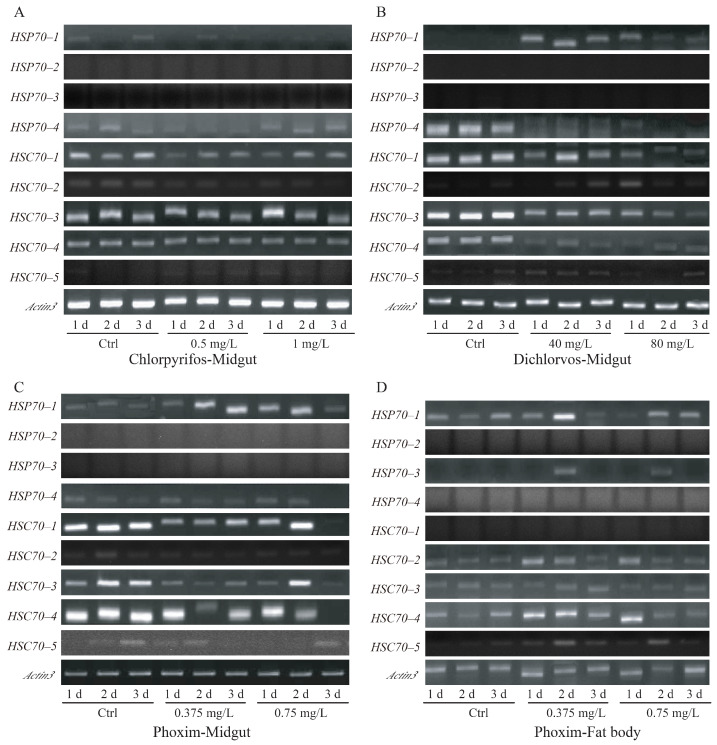
RT-PCR detection of response patterns for HSP70/HSC70 genes in the silkworm after exposure to three OPs. (**A**) Expressions in the midgut of the fifth-instar larvae after chlorpyrifos treatment. Two sub-lethal concentrations (0.5 mg/L and 1 mg/L) were used. (**B**) Responses of HSP70/HSC70 genes in the midgut of the silkworm under dichlorvos exposure. (**C**,**D**) Effects of phoxim exposure on the expressions of HSP70s/HSC70s in the midgut and fat body. Ctrl: control. The 1 d, 2 d, and 3 d mean 1 day, 2 days, and 3 days under OPs exposure, respectively.

Some studies suggested that HSP70s/HSC70s might prevent protein aggregates and promote the correct folding under pesticide stresses [29,40]. In this study, sub-lethal concentrations of three organophosphorus insecticides (dichlorvos, chlorpyrifos, and phoxim) were used as stressors. The important detoxification tissues (midgut and fat body) were used to detect the stress responses of HSP70/HSC70 genes in the fifth-instar larvae under OPs exposure for 24 h, 48 h, and 72 h (Figure 6). After exposure to chlorpyrifos, *HSP70–3* and *HSC70–1* were slightly inhibited in the midgut, while no obvious response was found for the other genes (Figure 6A). It was indicated that *HSP70–1* was induced by dichlorvos exposure (Figure 6B). However, *HSP70–4*, *HSC70–1*, *HSC70–3*, and *HSC70–4* were suppressed from the first day of treatment (Figure 6B). Under phoxim exposure, *HSP70–1* was slightly induced, and *HSC70–1*, *HSC70–3*, and *HSC70–4* were inhibited in the midgut (Figure 6C). In the fat body, *HSC70–4* showed slight induction under exposure to 0.375 mg/L phoxim. In general, most of the HSP70/HSC70 genes were often inhibited by pesticides, only *HSP70–1* and *HSC70–4* presented slight inductions in specific tissues or concentrations of pesticides. Furthermore, these results suggested that one gene may have different transcriptional responses in different tissues or under different OPs exposure.

## 4. Discussion

The heat shock proteins 70 belong to a ubiquitous family of molecular chaperones, which play essential roles in protein folding and are involved in protein refolding after a stress injury [44,45]. According to expression profiling and phylogenetic conservation, heat shock proteins 70 are classified as stress-inducible HSP70s and constitutively expressed HSC70s [1,8,44]. In this study, 9 genes were identified in the silkworm, which were divided into four HSP70s and five HSC70s. Relatively, gene numbers of HSC70s were comparable in the silkworm, *B. tabaci*, and *D. melanogaster*, and most of them presented 1:1:1 orthologous relationships (Figure 1). However, the stress-inducible HSP70s showed a greater number of genes to differentiate among the three species. For instance, four and ten members were found in *B. mori* and *B. tabaci*, respectively. These results suggested that duplication of HSP70s might play important roles in adapting specific niches. It is still worthy of further study.

In *B. tabaci*, expressions of all the HSP70/HSC70 genes were detected in the head and body part of the adults [13]. It was indicated that almost all the stress-inducible HSP70 genes presented quite low expressions in all the tissues of *B. tabaci* [13]. In this study, 9 HSP70/HSC70 genes were identified in the silkworm, and their spatio-temporal expression profiles were detected (Figure 3). Except for *HSC70–2*, the other 4 heat shock cognate 70 genes showed constitutively high expressions in various tissues of the fifth-instar larvae, pupae, and adults (Figure 3). For the stress-inducible HSP70 genes, they were specifically expressed in some tissues of the fifth-instar larvae (Figure 3A,D). However, *HSP70–2* and *HSP70–3* were also widely expressed in all the tissues of the pupae and adults (Figure 3B,C). Our results suggested that stress-inducible HSP70 genes might also play constitutive housekeeping roles in some developmental stages.

Previous studies indicated that a significant increase of heat shock protein 70 mRNA could be part of the protective mechanism to reduce cellular damage either in the testis lobe of *D. melanogaster* [24] or rat testis [46]. In the silkworm, almost all the HSP70/HSC70 genes were expressed in testis at all three developmental stages (Figure 3), which suggested that heat shock proteins 70 might play important roles in spermatogenesis.

Heat shock proteins are crucial for survival to acute thermal and cold stresses [10]. The molecular basis of HSP70/HSC70 chaperone activity stems from the ability of this protein to bind to misfolded or denatured proteins, thereby preventing their aggregation, which is harmful to cells [1,2,3,4]. Up-regulated expression of heat shock protein 72 was found in the silkworm following heat shock at 34 °C, 38 °C, and 42 °C, respectively [47]. Due to induced expression of the HSP70 gene, the cocoon and shell weight increased to 9.90 and 11.90%, respectively, compared with the control [47]. Thus, up-regulation of HSP70s/HSC70s should be one of the mechanisms for heat tolerance and could be used for improving vitality and fitness in organisms [48]. In this study, the expression responses of all the HSP70s were detected in the silkworm under thermal stress (Figure 4 and Figure 5). It was indicated that *HSP70–1*, *HSP70–2*, and *HSP70–3* were induced in the fat body, midgut, and silk gland. Induction response was also found for the *HSC70–1* in the midgut, *HSC70–4* in the fat body, and *HSC70–5* in the silk gland. After 2 °C cold exposure, *HSP70–1*, *HSP70–2*, and *HSP70–3* were significantly induced in multiple tissues. For the cognate forms, *HSC70–4* and *HSC70–5* were slightly up-regulated by cold stress. In general, expressions of HSP70 genes could be induced in multiple tissues at the same time, while HSC70 genes were induced only in one of the tissues and maybe in inhibited in other tissues. Similar results were also found in other organisms [13]. Thus, the inducible forms (HSP70s) might be more important in response to thermal and cold stresses.

During the detoxification of insecticides, a mass of reactive oxygen species are produced [40,49], and will further result in generating lipid hydroperoxides, such as the middle product phospholipid hydroperoxide and malondialdehyde (MDA) [26,40]. Those highly reactive electrophilic components could heavily damage proteins. HSP70s play important roles in promoting the correct folding of stress-accumulated proteins and preventing the formation of protein aggregates [29]. In *D. melanogaster*, after exposure to 0.15–1.5 ppb dichlorvos, expression of an HSP70 gene was significantly induced in the testes [24,26]. Multiple studies have shown that inducible responses of heat shock protein 70 genes could be detected after exposure to low concentrations of insecticides [31,50]. It was proposed that stress responses of HSP70/HSC70 genes can be used as a sensitive indicator for assessing the environmental burden of insecticides [31]. In this study, *HSP70–1* was induced at low and high concentrations of dichlorvos and phoxim exposure in the midgut of the silkworm (Figure 6B,C), and *HSC70–4* was slightly induced by 0.375 mg/L phoxim in the fat body (Figure 6D). This suggested that *HSP70–1* and *HSC70–4* might be related to promoting the correct folding of proteins during the insecticide stress in the silkworm. Interestingly, the stress response of a certain gene might be different in different tissues. For instance, *HSC70–4* was induced in the fat body, while it was inhibited in the midgut (Figure 6C,D). It was suggested that if HSP70s/HSC70s are used as biomarkers to monitor pesticide contamination, it is necessary to screen which genes and which tissues show induction responses in a targeted manner.

## 5. Conclusions

A total of 9 genes were identified in the silkworm, of which four stress-inducible forms (HSP70s) and five heat shock cognates (HSC70s) were classified. Most silkworm HSC70s showed orthologous relationships with *D. melanogaster* and *B. tabaci*, indicating that heat shock cognates were more conserved during evolution. In the silkworm, most of the HSC70 genes presented constitutive expressions in all the tissues and developmental stages. However, stress-inducible *HSP70–2* and *HSP70–3* also exhibit constitutive expression in the pupal and adult stages. Under thermal and cold stresses, the expressions of *HSP70–1*, *HSP70–2*, and *HSP70–3* were highly induced. *HSP70–1* could also be induced by dichlorvos and phoxim insecticides, respectively. Interestingly, the expression of *HSC70–4* was slightly induced under cold, thermal, and phoxim stresses. This study revealed valuable information about the heat shock proteins 70 in the silkworm and provided insights into environmental adaptation.

## Data Availability

The proteins sequences of the identified silkworm HSP70/HSC70 genes were included in Table S1. All published data are available upon formal request.

## Supplementary Materials

The following are available online at https://www.mdpi.com/article/10.3390/insects12100928/s1, Table S1: Sequence information of the HSP70s/HSC70s in the silkworm and two model species, Table S2: Primers of the silkworm HSP70/HSC70 genes used for RT-PCR and qPCR detection.
