# Supplementary material for: Heat Shock Protein 70 Family in Response to Multiple Abiotic Stresses in the Silkworm
Source: Insects. 2021 Oct 12;12(10):928. doi: 10.3390/insects12100928 (PMC8537551; doi:10.3390/insects12100928)
Supplement: Supplementary file 1 [file insects-12-00928-s001.zip › Table S2.pdf]

Table S2. Primers of the silkworm HSP70 genes used for RT-PCR and qPCR detection.

Gene	Primer sequence (5′ to 3′)	Amplification length (bp)	Annealing temperature (ºC)
HSP70–1	F: AAAGCCCAAAATCCAGATAGA	218	53
	R: TACGAAGCACATTCAGACCAG		
HSP70–2	F: CACTGTCGAAGAAGTTGATTGA	112	53
	R: CATTTGTATAAAGGTGGGGAGA		
HSP70–3	F: AGTGATGGACCCACAGTTGAG	174	53
	R: GTATTCACGGGCTGCTACAA		
HSP70–4	F: GTCATTCTGGTCGGAGGTAG	265	55
	R: GACAGGGTATAGATGTATTTCGT		
HSC70–1	F: GGCTCTACCCGTATCCCTAA	208	53
	R: TCACTCCACCGACAGTTTCA		
HSC70–2	F: AAGACTTTGATAGCCGAATG	209	51
	R: AGCCCTTGACAGTTTAGAATAG		
HSC70–3	F: CGGAATACATTTGGGTAACA	208	53
	R: ATCACAGTTCATTAGCCGTT		
HSC70–4	F: CAGGTCCACTCCGTCTTATGT	202	55
	R: CTTAGGTTTGCCTCCGTCACT		
HSC70–5	F: AGACGCTATGGCTATGCA	287	51
	R: GGACCTTAGGCATTCTC		
Actin3	F: TTTATTCTGAGCGTAAGTGG	205	51
	R: TAATGCTGGAACTCGTAATG		

Note: The GenBank accession number of *Actin3* gene is U49854.1. F: forward primer sequence; R: reverse primer sequence.
